# Exploring the possibility of omitting axillary surgery in patients with clinical node-positive breast cancer achieving ypT0 after neoadjuvant chemotherapy

**DOI:** 10.1007/s10549-025-07697-4

**Published:** 2025-04-12

**Authors:** Hideo Shigematsu, Momoko Takaya, Kanako Suzuki, Mutsumi Fujimoto, Haruka Ikejiri, Ai Amioka, Emiko Hiraoka, Shinsuke Sasada, Koji Arihiro, Morihito Okada

**Affiliations:** 1 Department of Surgical Oncology, Research Institute for Radiation Biology and Medicine, Hiroshima University, 1 - 2- 3-Kasumi, Minami-Ku, Hiroshima City, Hiroshima 734 - 8551 Japan; 2 Department of Anatomical Pathology, Hiroshima University Hospital, Hiroshima, 734 - 8551 Japan

**Keywords:** Breast cancer, Neoadjuvant chemotherapy, YpT0, YpN0, Axillary surgery omission

## Abstract

**Purpose:**

Axillary staging is commonly performed in patients with clinically node-positive (cN+) breast cancer undergoing neoadjuvant chemotherapy (NACT), regardless of pathological complete response (pCR). Recent evidence has suggested that ypT0 correlates with ypN0 and favorable prognosis, potentially supporting the omission of axillary staging in such cases. This study aimed to evaluate ypT0 as a predictive factor for ypN status and its prognostic significance in cN+ breast cancer treated with NACT.

**Methods:**

This retrospective study included 302 patients with cN+ breast cancer treated with NACT at Hiroshima University Hospital between 2006 and 2022. Patients were categorized into non-pCR, ypTis, or ypT0 based on ypT status. Associations between breast pCR, ypN status, recurrence-free survival (RFS), and overall survival (OS) were analyzed.

**Results:**

Among 302 patients (non-pCR, 74.2%; ypTis, 8.9%; ypT0, 16.9%), the ypN+ rates were 63.3%, 15.2%, and 3.9%, respectively. Logistic regression revealed significant associations among ypT0, ypTis, and ypN0. The five-year RFS and OS rates were 78.6% and 85.2% (non-pCR), 83.8% and 95.5% (ypTis), and 98.0% and 100.0% (ypT0), respectively. Cox regression identified ypT0, but not ypTis, as a significant prognostic factor for both RFS and OS.

**Conclusion:**

ypT0 status was associated with a low risk of ypN+ and favorable clinical outcomes in cN+ breast cancer, suggesting the potential feasibility of omitting axillary surgery in select patients.

## Introduction

Axillary lymph node dissection (ALND) is widely performed as the standard treatment for clinically node-positive (cN+) breast cancer [1]. However, this procedure is associated with complications such as upper arm lymphedema, postoperative pain, and restricted range of motion, which can significantly impact patients'quality of life [2, 3]. Recent advances in systemic therapy have increased rates of pathological complete response (pCR), particularly in human epidermal growth factor receptor 2 (HER2)-positive and triple-negative subtypes, raising the potential for de-escalating axillary surgery in selected patients.

Neoadjuvant chemotherapy (NACT) is now widely accepted as a standard treatment for early-stage breast cancer [4, 5]. Advances in systemic therapy have led to pCR rates exceeding 50% in highly chemotherapy-sensitive subtypes such as HER2-positive and triple-negative breast cancer [6–9]. In breast cancer treated with NACT, breast pCR is recognized as an independent predictor of axillary node negativity (ypN0) [10, 11]. Notably, patients with cN0 breast cancer achieving breast pCR have been shown to have a low risk of residual axillary disease [10, 12]. Consequently, clinical trials are currently underway to assess the feasibility of omitting axillary surgery in highly chemotherapy-sensitive cN0 breast cancer with a substantial response to NACT [13]. Conversely, in cN+ breast cancer, even when breast pCR is achieved, residual axillary disease (ypN+) has been reported in 11.3%–31.5% of cases [10, 12]. Therefore, further identification of low-risk cases is essential to facilitate the omission of axillary surgery in cN+ breast cancer responding significantly to NACT.

The definition of pCR generally includes the absence of invasive carcinoma and axillary lymph node metastases [14]. However, there is no consensus on whether residual ductal carcinoma in situ (ypTis) should be included in the definition of pCR [15–17]. From the perspective of predicting ypN0, it may be more appropriate to define pCR as ypT0, indicating the absence of both invasive and in situ carcinoma. A retrospective single-center study of cN+ breast cancer treated with NACT reported that the ypN0 rate was 94.3% in ypT0 breast cancer, compared to 58.8% in ypTis breast cancer [18]. These findings suggest the potential for omitting axillary surgery in ypT0 breast cancer. However, the study included a limited number of cN+ cases, highlighting the need for further investigation. Additionally, long-term prognostic data on ypT0 breast cancer are lacking, and the prognosis of cN+ breast cancer achieving ypT0 remains unclear.

This study aims to evaluate the utility of ypT0 as a predictor of ypN0 and its significance as a prognostic factor in cN+ breast cancer treated with NACT. The findings could have significant implications for minimizing surgical intervention and improving the quality of life for breast cancer patients.

## Materials and methods

### Study design

This retrospective observational study analyzed patients with cT1–4, N1–3, and M0 breast cancers who underwent NACT at the Hiroshima University Hospital between 2006 and 2022. This study included all cT4 breast cancer cases without subclassification into T4a–d. Diagnosis of cN+ status was confirmed through pathological examination via fine-needle aspiration or core-needle biopsy. Patients treated with NACT regimens other than anthracycline or taxane and those who did not undergo curative surgery for both the breast and axillary lymph nodes post-NACT were excluded. This study was approved by the Ethics Review Committee of Hiroshima University (approval number: E2018 - 1166). Given the retrospective nature of this study, which used hospital database records, the requirement for written informed consent was waived.

### Clinicopathological factors

The clinicopathological factors assessed included age at diagnosis, histological type, clinical TNM stage, tumor grade, hormone receptor (HR) status, HER2 status, post-NACT tumor (ypT) and nodal (ypN) status, NACT regimen, type of breast and axillary surgery, and adjuvant therapies for breast cancer. Clinical T (cT) and N (cN) stages were classified according to the TNM system [19]. HR positivity was defined as estrogen or progesterone receptor expression exceeding 1%, as assessed by immunohistochemistry [20]. HER2 status was evaluated using immunohistochemistry and/or in situ hybridization (ISH), with an immunohistochemistry score of 3+ or gene amplification by ISH indicating positivity [21]. Breast cancer was categorized into four subtypes: HR-positive/HER2-negative, HR-positive/HER2-positive, HR-negative/HER2-positive, and triple-negative. NACT typically included an anthracycline-and/or taxane-based regimen supplemented with trastuzumab and pertuzumab in HER2-positive cases. Pembrolizumab was not administered to triple-negative cases during this period. Surgical treatment choices for the breast and axillary regions were made at the discretion of the physicians. The ypT status was classified as non-pCR, ypTis, or ypT0. The ypN+ status indicated the presence of residual disease, including isolated tumor cells or micrometastases. Adjuvant capecitabine was administered after 2017 to patients with HER2-negative breast cancer who had residual invasive disease after NAC, following the results of the CREATE-X trial [22]. Radiation therapy protocols included treatment of the conserved breast after breast-conserving surgery, and the chest wall and regional lymph nodes. The postoperative follow-up consisted of regular physical examinations and annual mammography, with additional tests for symptoms. Breast cancer recurrence was identified clinically and/or via imaging with confirmatory pathological examinations, as necessary. Events involving contralateral or secondary cancers were not considered breast cancer recurrences. Recurrence-free survival (RFS) was calculated from the date of definitive surgery to either the date of diagnosis of recurrence or the last follow-up. Overall survival (OS) was determined from the date of definitive surgery to the date of death from any cause or last follow-up.

### Statistical analysis

Clinicopathological factors were analyzed as categorical variables. The association between these factors and breast pCR was assessed using the chi-square test. Logistic regression analysis was used to evaluate predictors of ypN0 status. The effect of breast pCR on RFS was analyzed using the log-rank test. Cox regression analysis was conducted to identify predictors of RFS and OS. Both odds and hazard ratios were reported with 95% confidence intervals (CIs). Statistical significance was set at *p* < 0.05. Statistical analyses were performed using the JMP software (version 18.0.1; JMP Statistical Discovery LLC, Cary, NC, USA).

## Results

### Patient characteristics

Between 2006 and 2022, a total of 526 patients with breast cancer underwent neoadjuvant chemotherapy (NACT). After excluding 210 patients with cN0 status, eight patients who received non-anthracycline or non-taxane NACT regimens, and six patients who did not undergo curative surgery post-NACT, 302 patients were eligible for the study. The patient demographics are summarized in Table 1. The median age was 56 years, and all participants were female. Invasive breast carcinoma of no special type was the predominant histology (97.7%). The most common clinical stages were cT2 (55.0%) and cN1 (70.5%). Subtype distribution included HR-positive/HER2-negative (52.3%), HR-positive/HER2-positive (17.6%), HR-negative/HER2-positive (10.6%), and triple-negative (19.5%).

**Table 1:** Patient characteristics

Factors	Total 302 (100)
Age, years	
Median 56 (interquartile range, 46–64)	
≤ 55	148 (49.0)
> 55	154 (51.0)
Sex	
Female	302 (100)
Histology	
Invasive breast carcinoma of no special type	295 (97.7)
Special type	7 (2.3)
Clinical T stage	
1	48 (15.9)
2	166 (55.0)
3	43 (14.2)
4	34 (14.9)
Clinical N stage	
1	213 (70.5)
2	44 (14.6)
3	45 (14.9)
Subtype	
HR-positive/HER2-negative	158 (52.3)
HR-positive/HER2-positive	53 (17.6)
HR-negative/HER2-positive	32 (10.6)
HR-negative/HER2-negative	59 (10.6)
Nuclear grade	
1	20 (6.6)
2	88 (29.1)
3	194 (64.2)
Neoadjuvant chemotherapy	
Anthracycline	5 (1.7)
Taxane	13 (4.3)
Anthracycline and taxane	284 (94.0)
Trastuzumab ± pertuzumab	82 (27.2)
Breast surgery	
Breast-conserving surgery	101 (33.4)
Mastectomy	201 (66.6)
Axillary surgery	
Sentinel lymph node biopsy	4 (1.3)
Axillary lymph node dissection	298 (98.7)
ypT status	
Non-pCR	224 (74.2)
ypTis	27 (8.9)
ypT0	51 (16.9)
Pathological nodal status after neoadjuvant chemotherapy	
ypN0	157 (52.0)
ypN+	145 (48.0)
Adjuvant chemotherapy	
Yes	76 (25.2)
No	226 (74.8)
Adjuvant radiotherapy	
Yes	240 (79.4)
No	62 (20.5)
Breast cancer recurrence	
Yes	61 (20.2)
No	241 (79.8)
Survival	
Alive	256 (84.8)
Death	46 (15.2)

Data are expressed as *n* (%), unless otherwise specified

HER2, human epidermal growth factor receptor 2; HR, hormone receptor; pCR, pathological complete response

Breast pCR rates were 16.9% for ypT0, 8.9% for ypTis, and 74.2% for non-pCR cases. Overall, 48.0% of patients were ypN+. Breast-conserving surgery was performed in 33.4%, and adjuvant radiation therapy was administered to 79.8% of patients. The median follow-up was 6.0 years (interquartile range: 2.8–9.9 years), during which 61 cases (20.2%) of breast cancer recurrence were documented. Recurrence sites included the conserved breast (2 cases), regional nodes (20 cases), bones (17 cases), lungs (20 cases), liver (16 cases), and brain (6 cases).

Table 2 shows the correlation between breast pCR and pre-NACT clinicopathological factors. A significant correlation was found between breast pCR and the breast cancer subtype, with a notably higher frequency of non-pCR in the HR-positive/HER2-negative subtype than in the other subtypes. A marginally significant correlation was observed between grade and breast pCR, particularly for stages cT3 and cT4.

**Table 2:** Association between breast pCR status and clinicopathological factors prior to neoadjuvant chemotherapy

Factors	Non-pCR (*n* = 218)	ypTis (*n* = 33)	ypT0 (*n* = 55)	*p* value
Age				
≤ 55 years	105 (71.0)	17 (11.5)	26 (17.6)	0.89
> 55 years	113 (73.4)	16 (10.4)	25 (16.2)	
Clinical T stage				
1, 2	147 (68.7)	25 (11.7)	42 (19.6)	0.087
3, 4	71 (80.7)	8 (9.1)	9 (10.2)	
Clinical N stage				
1	155 (72.8)	25 (11.7)	33 (15.5)	0.52
2, 3	63 (70.8)	8 (9.0)	18 (20.2)	
Subtype				
HR-positive/HER2-negative	139 (88.0)	8 (5.1)	11 (7.0)	< 0.001
HR-positive/HER2-positive	27 (50.9)	14 (26.4)	12 (22.6)	
HR-negative/HER2-positive	11 (34.4)	7 (21.9)	14 (43.8)	
HR-negative/HER2-negative	41 (69.5)	4 (6.8)	14 (23.7)	
Nuclear grade				
1, 2	82 (75.9)	15 (13.9)	11 (10.2)	0.046
3	136 (70.1)	18 (9.3)	40 (20.6)	

Data are expressed as *n* (%) unless otherwise specified

HR, hormone receptor; HER2, human epidermal growth factor receptor 2; pCR, pathological complete response

### Breast pCR and ypN status

Table 3 shows the ypN status according to breast pCR across all cases and within each breast cancer subtype. Overall, the frequency of ypN+ was 63.3% for non-pCR, 15.2% for ypTis, and 3.9% for ypT0 cases. Within the HR-positive/HER2-negative subtype, the frequency of ypN+ was 71.9% for non-pCR, 12.5% for ypTis, and 18.2% for ypT0 cases. The ypN+ frequencies for ypTis in the HR-positive/HER2-positive, HR-negative/HER2-positive, and HR-negative/HER2-negative subtypes were 7.1%, 14.3%, and 50.0%, respectively, whereas no ypN+ cases were noted for ypT0. Table 4 shows the associations between clinicopathological factors and ypN+ status in both univariate and multivariate analyses. Multivariate analysis revealed that both ypT0 (odds ratio 0.03; 95% CI: 0.007–0.13; *p* < 0.001) and ypTis (odds ratio 0.15; 95% CI 0.05–0.43; *p* < 0.001) significantly predicted the ypN0 status. In addition to breast pCR, cN1 stage and non-HR-positive/HER2-negative subtypes were significantly associated with ypN0.

**Table 3:** Pathologic nodal status according to breast pCR stratified by breast cancer subtype

Breast cancer subtype	ypN0	ypN+
All subtypes		
Non-pCR	80 (36.7)	138 (63.3)
ypTis	28 (84.9)	5 (15.2)
ypT0	49 (96.1)	2 (3.9)
HR-positive/HER2-negative		
Non-pCR	39 (28.1)	100 (71.9)
ypTis	7 (87.5)	1 (12.5)
ypT0	9 (81.8)	2 (18.2)
HR-positive/HER2-positive		
Non-pCR	14 (51.9)	13 (48.2)
ypTis	13 (92.9)	1 (7.1)
ypT0	12 (100)	0 (0)
HR-negative/HER2-positive		
Non-pCR	7 (63.6)	4 (36.4)
ypTis	6 (85.7)	1 (14.3)
ypT0	14 (100)	0 (0)
HR-negative/HER2-negative		
Non-pCR	20 (48.8)	21 (51.2)
ypTis	2 (50.0)	2 (50.0)
ypT0	14 (100)	0 (0)

Data are expressed as *n* (%) unless otherwise specified

HR, hormone receptor; HER2, human epidermal growth factor receptor 2; pCR, pathological complete response

**Table 4:** Association between clinicopathological factors and ypN+ in univariate and multivariate analyses

Variables	Univariate			Multivariate		
	Odds ratio	95% CI	*p* value	Odds ratio	95% CI	*p* value
Age, years						
≤ 55	Ref		0.35	1		0.77
> 55	1.24	0.79–1.95		1.09	0.62–1.89	
Clinical T stage						
cT1, 2	1		0.15	1		0.72
cT3, 4	1.45	0.88–2.39		1.12	0.60–2.08	
Clinical N stage						
cN1	1		0.019	1		0.003
cN2, 3	1.81	1.10–3.01		2.73	1.40–5.35	
Subtype						
HR-positive/HER2-negative	1			1		
HR-positive/HER2-positive	0.19	0.09–0.38	< 0.001	0.31	0.14–0.70	0.005
HR-negative/HER2-positive	0.10	0.03–0.25	< 0.001	0.25	0.08–0.79	0.018
HR-negative/HER2-positive	0.34	0.18–0.63	< 0.001	0.41	0.20–0.85	0.016
Grade						
1, 2	1		0.22	1		0.96
3	0.74	0.46–1.19		0.99	0.55–1.76	
Breast pCR						
Non-pCR	1			1		
ypTis	0.10	0.03–0.26	< 0.001	0.15	0.05–0.43	< 0.001
ypT0	0.02	0.004–0.08	< 0.001	0.03	0.007–0.13	< 0.001

CI, confidence interval; HR, hormone receptor; HER2, Human epidermal growth factor receptor 2; pCR, pathological complete response

### Breast pCR and prognosis

**Fig. 1:**
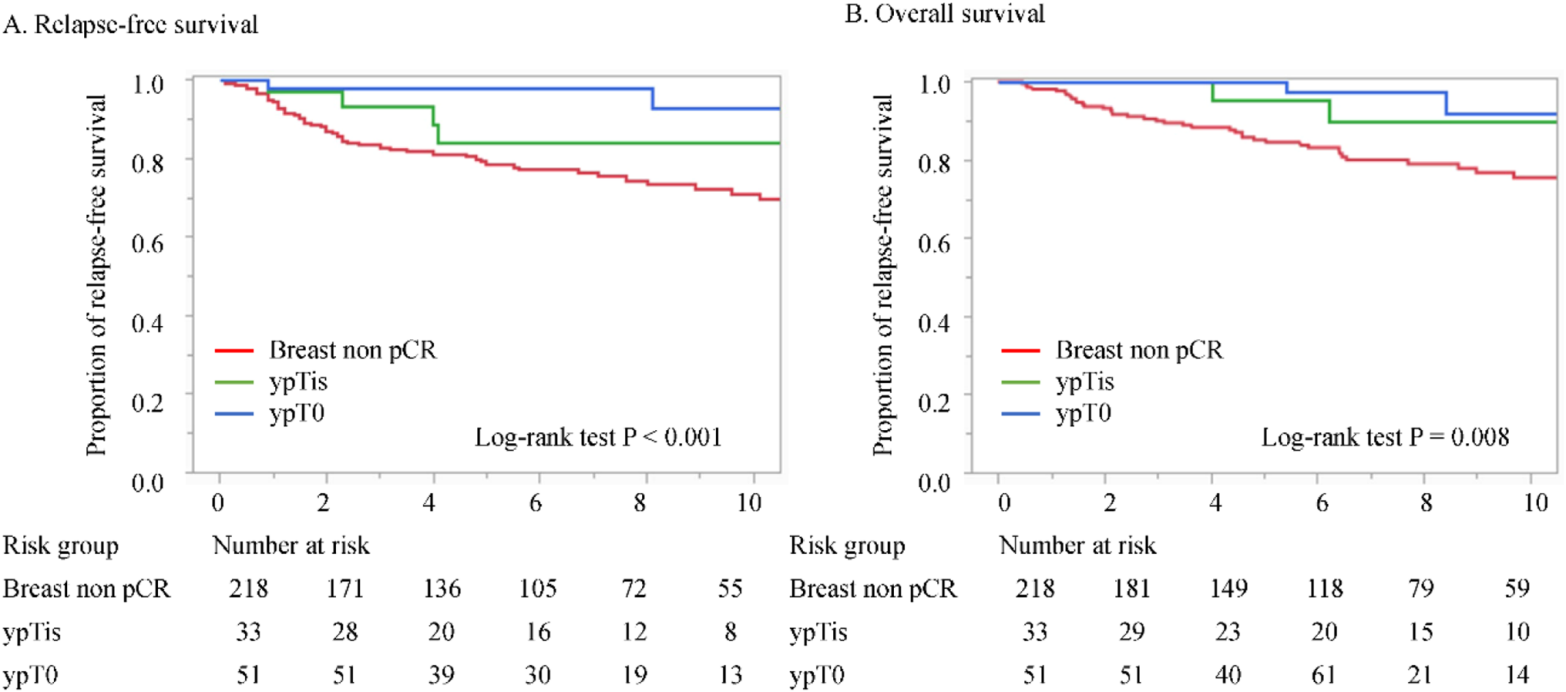
Kaplan–Meier survival curves for **A** relapse-free survival and **B** overall survival according to the ypT status. The survival curves were compared using the log-rank test. pCR, pathological complete response

Figure 1 shows the Kaplan–Meier survival curves for RFS and OS, stratified by breast pCR. Breast cancer recurrence was documented in 55 patients without pCR, four patients with ypTis, and two patients with ypT0. The five-year RFS rates were 98.0%, 83.8%, and 78.6% for ypT0, ypTis, and those without pCR, respectively (*p* = 0.0018). Deaths were recorded in 42 patients without pCR, two with ypTis, and three with ypT0. The five-year OS rates were 100.0% for ypT0, 95.5% for ypTis, and 85.2% for those without pCR (*p* = 0.015). Table 5 summarizes the results of the Cox regression analysis for RFS and OS. ypT0 was identified as a significant favorable prognostic factor for RFS (hazard ratio 0.19, 95% CI 0.04–0.83, *p* = 0.027) and OS (hazard ratio 0.23, 95% CI 0.06–0.83, *p* = 0.025). However, ypTis was not a significant factor. Other significant predictors of poor RFS included hormone receptor-negative/HER2-negative status (*p* = 0.001), cT3/4 (*p* = 0.020), and cN2/3 (*p* = 0.050). In contrast, significant predictors of deterioration in OS included hormone receptor-negative/HER2-negative status (*p* = 0.007) and cT3/4 (*p* = 0.049).

**Table 5:** Cox regression analysis of (A) recurrence-free survival and (B) overall survival in patients with cN + breast cancer

A. Relapse-free survival						
Variables	Univariate analysis			Multivariate analysis		
	Hazard ratio	95% CI	*p* value	Hazard ratio	95% CI	*p* value
Age						
≤ 55	1		0.60	1		0.23
> 55	0.87	0.53–1.45		0.73	0.43–1.23	
Clinical T stage						
cT1, 2	1		0.006	1		0.020
cT3, 4	2.05	1.23–3.43		1.91	1.10–3.33	
Clinical N stage						
cN1	1		0.003	1		0.050
cN2, 3	2.17	1.31–3.62		1.73	1.00–3.01	
Subtype						
HR-positive/HER2-negative	1			1		
HR-positive/HER2-positive	0.77	0.36–1.68	0.51	1.09	0.48–2.49	0.84
HR-negative/HER2-positive	0.29	0.07–1.21	0.090	0.69	0.15–3.09	0.62
HR-negative/HER2-positive	1.95	1.10–3.44	0.02	2.78	1.49–5.19	0.001
Grade						
1, 2	1		0.25	1		0.41
3	1.38	0.80–2.40		1.28	0.71–2.29	
Breast pCR						
Non-pCR	1			1		
ypTis	0.46	0.18–1.28	0.14	0.61	0.21–1.77	0.36
ypT0	0.13	0.03–0.54	0.005	0.19	0.04–0.83	0.027
pN status						
ypN0	1		0.002	1		0.059
ypN+	2.75	1.61–4.70		1.81	0.98–3.36	

B. Overall survival						
	Univariate analysis			Multivariate analysis		
Variables	Hazard ratio	95% CI	*p* value	Hazard ratio	95% CI	*p* value
Age, years						
≤ 55	1		0.87	1		0.71
> 55	1.05	0.59–1.86		0.90	0.50–1.61	
Clinical T stage						
cT1, 2	1		0.014	1		0.049
cT3, 4	2.09	1.16–3.74		1.89	1.00–3.55	
Clinical N stage						
cN1	1		0.014	1		0.071
cN2, 3	2.07	1.16–3.69		1.80	0.95–3.42	
Subtype						
HR-positive/HER2-negative	1			1		
HR-positive/HER2-positive	0.64	0.24–1.67	0.36	0.88	0.32–2.42	0.81
HR-negative/HER2-positive	0.77	0.27–2.26	0.63	1.58	0.49–5.10	0.44
HR-negative/HER2-positive	1.89	0.98–3.66	0.058	2.74	1.32–5.66	0.007
Grade						
1, 2	1		0.40	1		0.57
3	1.31	0.70–2.45		1.21	0.63–2.35	
Breast pCR						
Non-pCR	1			1		
ypTis	0.30	0.07–1.24	0.10	0.31	0.07–1.36	0.12
ypT0	0.26	0.08–0.85	0.026	0.23	0.06–0.83	0.025
pN status						
ypN0	1		0.014	1		0.49
ypN+	2.07	1.16–3.69		1.27	0.64–2.52	

CI, confidence interval; HR, hormone receptor; HER2, Human epidermal growth factor receptor 2; pCR, pathological complete response

## Discussion

This study demonstrated that ypT0 is a strong predictor of ypN0 status and favorable prognosis in cN+ breast cancer patients treated with neoadjuvant chemotherapy (NACT). The ypN+ rate was significantly lower in ypT0 cases compared to ypTis and non-pCR, and five-year recurrence-free survival (RFS) and overall survival (OS) rates were highest in ypT0 cases. These findings suggest that ypT0 status could guide the omission of axillary surgery in selected patients.

pCR serves as a surrogate marker for a favorable prognosis and is regarded as one of the primary endpoints of NACT [17]. Therefore, pCR was defined as the presence of both breast pCR and ypN0 in this study. However, the definitions of breast pCR vary across research groups, with ypT0 or ypT0/ypTis being commonly used, although studies have indicated that ypTis breast cancer may have a higher risk of local recurrence and poorer prognosis compared with ypT0 breast cancer [16, 23]. Consequently, categorizing breast pCR into ypT0 and ypTis may allow for a more precise evaluation of response to NACT.

Additionally, defining breast pCR as ypT0 in patients with cN+ breast cancer undergoing NACT has proven effective in accurately predicting ypN0. A study utilizing the National Cancer Database identified breast pCR as ypT0/Tis, with observed ypN+ frequencies of 12.4% and 14.1% in patients with HER2-positive and HR-negative/HER2-negative breast cancer, respectively [12]. Conversely, a report from the MD Anderson Cancer Center, which defined breast pCR solely as ypT0, noted ypN+ frequencies of 11.9% and 8.6% in similar cohorts [10]. Furthermore, a retrospective analysis from a single institution suggested that ypT0 breast cancers exhibited a lower frequency of ypN+ than ypTis cancers [18]. Therefore, our study further explored the predictive accuracy of ypT0 and ypTis for the ypN0 status in patients with cN+ breast cancer treated with preoperative chemotherapy and found a markedly low risk of ypN+ in ypT0 cancers, whereas ypTis cancers showed a certain incidence of ypN+. Moreover, consistent with previous studies, cN1 and non-HR-positive/HER2-negative subtypes were confirmed as predictive factors for ypN0. These findings imply that patients with HR-negative or HER2-positive cN1 breast cancer who achieve ypT0 may be suitable candidates for omission of axillary surgery because of their low risk of residual axillary disease.

The classification of pCR into ypT0 and ypTis in patients with cN+ breast cancer undergoing NACT has been shown to improve the accuracy of prognostic predictions [16, 23]. A retrospective observational study involving 2066 patients with ypT0/is ypN0 from five neoadjuvant GBG/AGO-B trials reported the 4-year disease-free survival, distant disease-free survival, and OS for ypT0 and ypTis breast cancers to be 90.8%, 93.0%, and 95.4%, respectively, compared to 84.5%, 90.5%, and 94.8%, respectively, for ypTis breast cancer, demonstrating a trend toward a better prognosis in ypT0 breast cancer [23]. This trend was particularly pronounced in HER2-positive or HR-negative HER2-negative subtypes. Similarly, in the current study, which focused on cN+ breast cancer, ypT0 was correlated with a favorable prognosis and was identified as an independent prognostic factor in multivariate analysis. Conversely, ypTis breast cancer exhibited a certain recurrence rate and was not considered a significant prognostic factor. The improved prognosis associated with ypT0 breast cancer may stem from the reduced risk of ypN+ and the potential control of micrometastases through the elimination of intraductal lesions. Therefore, from the viewpoint of prognostic prediction, ypT0 accurately reflects the therapeutic effects of NACT.

Moreover, recent studies have challenged the significance of axillary staging in the management of occult axillary diseases. Axillary lymph node metastases are detected in over 10% of cases of low-risk cN0 breast cancer; however, omitting axillary staging does not compromise local control or long-term prognosis [24]. Additionally, while non-sentinel lymph node metastases are found in 30%–40% of breast cancers with sentinel lymph node metastases, further axillary dissection does not adversely affect prognosis [25, 26]. These results suggest that the detection of occult axillary disease does not influence the local control or long-term outcomes. Therefore, together with previous findings, our study indicates that ypT0 in patients with cN+ breast cancer treated with NACT correlates with a low risk of residual axillary disease and a favorable prognosis.

De-escalation of axillary surgery for cN+ breast cancer following neoadjuvant NACT has been under investigation, and several clinical trials, including Alliance Z1071, SENTINA, and SN-FNAC, have evaluated the feasibility of sentinel lymph node biopsy (SNB) in this setting [27–29]. These studies have reported a relatively high false-negative rate and a decrease in sentinel lymph node identification rate after NACT, indicating the need for careful patient selection. In our study, a low frequency of residual axillary disease was observed in cases achieving ypT0. This finding suggests that SNB-based de-escalation of axillary surgery may be applicable in ypT0 cases, and in the future, this approach may further evolve toward minimizing axillary surgery, ultimately leading to omission of axillary surgery.

Nonetheless, this study has some limitations. Its single-center, retrospective, observational design makes it susceptible to selection bias and missing data. Additionally, the small number of cases limits the sample size for various breast cancer subtypes and patients achieving breast pCR or ypN0. This raises concerns that the results of these subgroup analyses may have been due to chance. Furthermore, this study included all cT4 breast cancer cases without subclassification into T4a–d. Given its poor prognosis, T4 d is often excluded from studies evaluating axillary surgery de-escalation. Therefore, careful interpretation of our findings is warranted, and future research should consider a more detailed classification of T4 disease. Therefore, it is necessary to verify our results in larger cohorts, utilizing large-scale databases or clinical trial data to validate the prognostic value of ypT0 as a predictor of ypN0 and long-term outcomes and overcome these limitations.

## Conclusions

In this study, we identified ypT0 as a strong predictor of ypN0 and favorable prognosis in cN+ breast cancer patients treated with neoadjuvant chemotherapy. This finding highlights the potential to omit axillary surgery in selected patients. Furthermore, integrating ypT0 into clinical practice could advance personalized treatment strategies, particularly for HER2-positive and triple-negative subtypes. Future studies are warranted to validate these findings and explore their broader applicability.

## Data Availability

The datasets used during the current study are available from the corresponding author upon reasonable request.
